# A very low-carbohydrate diabetes prevention program for veterans with prediabetes: a single-arm mixed methods pilot study

**DOI:** 10.3389/fnut.2023.1069266

**Published:** 2023-05-17

**Authors:** Dina H. Griauzde, Cheryl Hershey, Jamie Michaels, Richard R. Evans, Caroline R. Richardson, Michele Heisler, Jeffrey T. Kullgren, Laura R. Saslow

**Affiliations:** ^1^VA Ann Arbor Healthcare System, Ann Arbor, MI, United States; ^2^Department of Internal Medicine, University of Michigan Medical School, Ann Arbor, MI, United States; ^3^University of Michigan Institute for Healthcare Policy and Innovation, Ann Arbor, MI, United States; ^4^Department of Family Medicine, University of Michigan Medical School, Ann Arbor, MI, United States; ^5^Department of Health Behavior and Health Education, School of Public Health, University of Michigan, Ann Arbor, MI, United States; ^6^Department of Health Management and Policy, University of Michigan School of Public Health, Ann Arbor, MI, United States; ^7^University of Michigan School of Nursing, Ann Arbor, MI, United States

**Keywords:** prediabetes, diabetes prevention, low-carbohydrate, weight loss, Veterans

## Abstract

**Introduction:**

All Veterans Affairs (VA) Medical Centers offer the MOVE! Weight Management Program to help patients achieve and maintain a healthy weight through a calorie-restricted, low-fat diet and increased physical activity. Yet, most MOVE! participants do not achieve clinically significant weight loss of ≥5%. A carbohydrate-restricted diet may help more Veterans to achieve ≥5% weight loss.

**Methods:**

This was a single-arm explanatory sequential mixed methods pilot study conducted in one VA health care system. Veterans with prediabetes and body mass index ≥25 kg/m2 were invited to participate in a group-based, virtual, very low-carbohydrate Diabetes Prevention Program (VLC-DPP) consisting of 23 sessions over 12 months. Participants were taught to follow a very low-carbohydrate eating pattern, defined as 20–35 grams of net carbohydrates per day. The primary outcomes were measures of feasibility and acceptability, including program uptake and session attendance. Secondary outcomes included change in weight, hemoglobin A1c, lipids, and patient-reported measures of food cravings, stress eating, perceived health status, and motivation. Interviews were conducted at 6 months to identify factors that facilitated or hindered participants’ achievement of ≥5% weight loss.

**Results:**

Among 108 screened Veterans, 21 enrolled in the study (19%), and 18 were included in the analytic cohort. On average, participants attended 12.4/16 weekly sessions and 3.6/8 bimonthly or monthly sessions. At 12 months, mean percent weight loss was 9.4% (SD = 10.7) with 9 participants (50%) achieving ≥5% weight loss. Three factors facilitated achievement of ≥5% weight loss among 10/16 interviewees: (1) enjoyment of low-carbohydrate foods; (2) careful monitoring of carbohydrate intake; and (3) reduced hunger and food cravings. Three factors hindered achievement of ≥5% weight loss among 6/16 interviewees: (1) food cravings, particularly for sweets; (2) challenges with maintaining a food log; and (3) difficulty with meal planning.

**Conclusion:**

A VLC-DPP is feasible and acceptable and shows preliminary efficacy among Veterans with prediabetes. The program’s weight loss effectiveness compared to standard MOVE! should be evaluated in a larger-scale trial. Such a program may be offered in addition to the standard MOVE! program to expand the menu of evidence-based lifestyle counseling options for Veterans.

**Clinical Trial Registration:**

https://clinicaltrials.gov/ct2/show/NCT04881890, identifier NCT04881890.

## 1. Introduction

Veterans who receive care within the Veterans Health Administration (VHA) are disproportionately burdened by obesity and its cardiometabolic consequences compared to non-Veteran populations. Approximately 25% of Veterans have type 2 diabetes mellitus–more than double the prevalence among non-Veterans–and an additional one-third are estimated to have prediabetes (1–3). Excess body weight is a primary risk factor for metabolic dysregulation and hyperglycemia (4). Fortunately, weight loss can help patients prevent, control, and often reverse cardiometabolic conditions. As little as 5% weight loss can improve blood glucose, insulin, and triglyceride levels (5), and prevent progression to type 2 diabetes among individuals with prediabetes (6).

Since 2001, the VHA’s National Center for Health Promotion and Disease Prevention has prioritized treatment of overweight and obesity through the development, implementation, and dissemination of the MOVE! Weight Management Program (referred to hereafter as MOVE!) (7). MOVE! is a lifestyle change program based on the Center for Disease Control and Prevention’s (CDC’s) National Diabetes Prevention Program (NDPP) (8). All Veterans Affairs (VA) Medical Centers offer MOVE! (9), which consists of individual or group-based lifestyle change sessions delivered in-person or remotely by telephone or video. MOVE! encourages participants to follow a calorie-restricted eating pattern and to engage in increased levels of physical activity. Despite the widespread availability of MOVE!, rates of participation remain low (10% among eligible Veterans) and most participants (≥ 75%) do not achieve ≥5% weight loss within 12 months (10, 11). Thus, while MOVE! provides an important foundation for weight management within the VHA, effective strategies that build on this foundation are needed to further engage and support weight loss among Veterans with obesity.

The VA/Department of Defense (DoD) Clinical Practice Guideline (CPG) for the Management of Adult Overweight and Obesity recommends the use of preference-sensitive dietary change approaches (12, 13), as the effect of any specific diet on weight loss varies markedly between individuals (14) and diet choice may play a key role in engaging individuals in weight management treatment (15–17). Low-carbohydrate diets consisting of foods such as meat, poultry, fish, eggs, tofu, tempeh, nuts, seeds, leafy greens, and other non-starchy vegetables are one evidence-based dietary approach with known appeal among Veterans (18). Low- and very low-carbohydrate diets–commonly defined as 10–26% and < 10% total daily energy from carbohydrate, respectively–can support weight loss, glycemic control, and favorable changes in cholesterol, blood pressure, and self-reported measures of energy, hunger, and food cravings while reducing the need for medications to control chronic conditions (19–22). In a randomized controlled trial (RCT) testing the role of diet choice among Veterans with obesity, Veterans demonstrated a preference for low-carbohydrate over low-fat diets (58% vs. 42%), and both diets supported mean percent weight loss ≥5% (18). Accordingly, a low-carbohydrate lifestyle change program may be one promising strategy to expand the menu of evidence-based weight management treatment options for Veterans with overweight and obesity.

Our team previously developed and pilot tested a very low-carbohydrate Diabetes Prevention Program (VLC-DPP) among a non-Veteran population with prediabetes and found the program to be feasible and acceptable to patients (23). The present study has two key objectives. The first objective is to evaluate the feasibility and acceptability of a VLC-DPP among Veterans with prediabetes. The second objective is to evaluate mean change in body weight and hemoglobin A1c (HbA1c) at 6 and 12 months. We hypothesized that a VLC-DPP would be feasible, acceptable, and effective among Veterans with prediabetes. These data will inform future initiatives to rigorously evaluate preference-sensitive weight management treatment within the VHA and help more Veterans achieve ≥5% weight loss.

## 2. Materials and methods

### 2.1. Study design

We conducted a single-arm explanatory sequential mixed methods (24) pilot study to examine the feasibility, acceptability, and preliminary effectiveness of a VLC-DPP among Veterans with prediabetes. We examined change in weight, hemoglobin A1c (HbA1c), and patient-reported measures of food cravings, stress eating, perceived health status, and motivation to prevent type 2 diabetes over the 12-month study period. Qualitative interviews were conducted at 6 months to explore participants’ experiences with the program and strengthen our understanding of quantitative findings (25). The study was approved by the VA Ann Arbor Healthcare System (VAAAHS) Institutional Review Board and conducted from October 2020 to September 2021. Trial registration: clinicaltrials.gov, NCT04881890. Registered May 11, 2021, https://clinicaltrials.gov/ct2/show/NCT04881890.

### 2.2. Setting

The intervention was intended to be delivered through in-person, group-based sessions. However, due to the COVID-19 pandemic, the intervention was delivered remotely using the VA Video Connect platform (26). VA Video Connect allows for secure virtual visits with Veterans and their care team; it can accommodate multiple simultaneous video feeds to allow for group interactions, and it can be accessed from any computer, tablet, or mobile device (e.g., Smartphone) with internet connection. To better facilitate group interaction on a virtual platform, we offered two small, closed groups rather than a single in-person group. At baseline, participants self-selected group participation on either Monday afternoons or Wednesday mornings.

### 2.3. Participants and recruitment

The intervention was conducted among individuals who received primary care within the VAAAHS, which includes the Lieutenant Colonel Charles S. Kettles VA Medical Center (VAMC) and six other freestanding VA outpatient health clinics serving a diverse patient population throughout southeast Michigan and Toledo, Ohio (27). Study inclusion criteria were: (a) Overweight, defined as body mass index (BMI) ≥ 25 kg/m^2^; (b) prediabetes, defined as HbA1c ≥ 5.7% and ≤ 6.4% drawn within 6 months of the anticipated study start date; (c) willingness and ability to participate in group-based, online classes using video; (d) ability to engage in at least light physical activity; and (e) willingness to self-weigh at least once weekly and report these data to the study team. Individuals without access to video-enabled technology or a home scale were provided with a webcam and/or scale by the study team. Study exclusion criteria were: (a) history of type 1 diabetes; (b) use of anti-hyperglycemic medications other than metformin; (c) current participation in another lifestyle or behavior change program or research study; (d) use of anti-obesity medications; (e) adherence to a vegetarian or vegan dietary pattern; (f) inability to read, write, or speak English; (g) inability to provide informed consent; or (h) pregnant or intention to become pregnant during the intervention period.

Potentially eligible Veterans were identified using VA Health Services Research & Development Corporate Data Warehouse. Study invitation letters were sent to 167 individuals. The letters briefly described the intervention and provided individuals with study team contact information if they desired to enroll and/or learn more about the study. Approximately 2 weeks after letters were sent, study staff reached out to Veterans by telephone to further describe the study, answer questions, and screen interested Veterans for eligibility. Interested and eligible Veterans completed written informed consent, a baseline survey, and laboratory tests drawn at the Lieutenant Colonel Charles S. Kettles VAMC or another freestanding VA outpatient health clinic within the VAAAHS. For participants who chose to have laboratory tests obtained at the Lieutenant Colonel Charles S. Kettles VAMC, a study team member (CH) met participants to complete written informed consent and the baseline survey prior to participants’ blood draw. For participants who chose to have laboratory tests obtained at another VA outpatient health clinic, written informed consent and the baseline survey were sent by postal mail and completed prior to laboratory testing; a study team member (CH) was available by phone to answer questions during the consent process.

### 2.4. Intervention

Our team previously developed and pilot tested a very low-carbohydrate adaptation of the CDC’s NDPP among a non-Veteran population (23). The CDC’s NDPP is similar to MOVE! in structure and content, and prior work has demonstrated similar 12-month weight change outcomes (8). Like MOVE!, the NDPP teaches participants to follow a low-fat, calorie-restricted eating pattern and to engage in at least 150 min of moderate intensity physical activity per week with the goal of achieving and maintaining 5 to 7% body weight loss (28).

A detailed summary of VLC-DPP intervention components is reported using the Template for Intervention Description and Replication (TIDieR) checklist in Appendix 1 (29). In brief, we substantially adapted the content of the NDPP’s dietary change sessions to teach participants to follow a very low-carbohydrate eating pattern rather than a low-fat, calorie-restricted eating pattern. We minimally altered NDPP sessions focused on other topics such as physical activity or behavior change. Appendix 2 shows NDPP and VLC-DPP session topics and denotes sessions with substantial versus minimal content modifications. Both programs consist of 16-weekly sessions delivered over 6 months (i.e., core phase) followed by 7 bimonthly or monthly sessions (i.e., maintenance phase) (30).

VLC-DPP participants were taught to consume 20–35 grams of net carbohydrates per day, defined as total grams of carbohydrate minus grams of dietary fiber. Participants were not provided with an explicit calorie limit or protein intake goal. Rather, participants were encouraged to eat when hungry and stop when satisfied, and they were instructed to keep protein intake similar to baseline. Participants were taught to change one meal per week from a high- to a very low-carbohydrate option, starting with breakfast and aiming for approximately 10 grams of net carbohydrates per meal or less. Participants were encouraged to consume foods such as meat, fish, poultry, eggs, cheese, seeds, nuts, leafy greens, non-starchy vegetables, and some fruits (e.g., berries). Participants who preferred plant-based sources of protein were encouraged to consume tofu and tempeh. Participants were taught to use low-carbohydrate substitutes in place of common high carbohydrate foods (e.g., cauliflower rice in place of grain rice).

Participants were encouraged to explore a variety of low-carbohydrate foods that appealed to their preferences and budget constraints. Participants were not provided with specific meal plans but rather provided with a variety of low-carbohydrate alternatives to high-carbohydrate foods and meals. For example, low-carbohydrate breakfast options included scrambled eggs, an omelet with broccoli and cheese, full-fat unsweetened Greek yogurt with pecans, a crustless quiche, low-carbohydrate pancakes, waffles or muffins, or a low-carbohydrate shake. Participants were taught to search for recipes online using search teams like “low-carb pancakes” and reviewing nutrition facts to select options with approximately 5 grams of net carbohydrates per serving. Participants were encouraged to share their favorite low-carbohydrate foods and meals with classmates during group sessions.

During the maintenance phase, participants were advised that they could maintain their very low-carbohydrate eating pattern or they could gradually liberalize their daily carbohydrate intake. Participants desiring to add carbohydrates to their eating plan were advised to increase their daily carbohydrate intake by 5 grams of net carbohydrates and maintain that change for at least 1 week. For example, individuals consuming 30 grams of net carbohydrate per day during the core phase were advised to increase to 35 grams of net carbohydrate per day and maintain that level for at least 1 week before making another change. Participants were advised to continue to self-weigh and to use increases in body weight, hunger, or food cravings as an indication that the carbohydrate intake was too high. In contrast, weight stability or continued weight loss without excessive hunger or cravings would indicate a well-tolerated carbohydrate level.

The intervention was delivered by a VA Registered Dietician with certification in diabetes education. She was trained to deliver low-carbohydrate education through self-guided review of the program curriculum and selected online resources (31). A primary care provider (PCP) with training in obesity medicine and experience with low-carbohydrate nutrition counseling (the principal investigator: DHG) provided additional training, as needed, and was present at all sessions to assist with answering participants’ questions, if necessary.

PCPs were notified of their patient’s participation in the study through electronic health record messaging. PCPs were also notified of participants’ weight changes and laboratory results at 6 and 12 months. Additional communication with PCPs occurred as needed. For example, when participants occasionally disclosed medical or psychosocial needs to a study team member, this information was communicated to PCPs to facilitate appropriate care for individual participants.

### 2.5. Data collection

Potentially eligible study participants were identified by HbA1c within the prediabetes range drawn within 6 months of the program’s anticipated start date. After providing written informed consent, individuals were asked to complete baseline laboratory and survey measures. Laboratory measures included HbA1c and fasting lipids. Individuals were not excluded from study participation if their baseline HbA1c was outside the prediabetes range (<5.7% or > 6.4%) provided they met other study criteria. These laboratory measures were repeated at 6 and 12 months.

At baseline, 6, and 12 months, participants were invited to complete a paper survey. A study team member (CH) met participants to complete surveys on the day of laboratory testing, if testing was obtained at the Lieutenant Colonel Charles S. Kettles VAMC. If laboratory tests were obtained at another VA outpatient health clinic, surveys were sent by postal mail. Survey responses were subsequently entered into REDCap (32) by a study team member. At baseline, participants were asked to report demographic and socio-economic information. At each time point, we used validated survey measures to assess key patient-centered outcomes. Specifically, food cravings were assessed using the 8-item Control of Eating Questionnaire subscales for savory and sweet food cravings (33). Stress eating was assessed using the 4-item coping subscale of the Palatable Eating Motives Scale (34), and a 2-item assessment of participants’ usual response to a stressful event (35). Patient-reported physical and mental health was assessed using the 10-item Patient-Reported Outcomes Measurement Information System Global Health survey (36). Sources of motivation to prevent type 2 diabetes (i.e., intrinsic vs. extrinsic) were assessed using the 15-item Treatment Self-Regulation Questionnaire (37). Survey items, response options, and scoring details are shown in Appendix 3.

To evaluate possible side effects from a very low carbohydrate diet, we also assessed self-reported physical symptoms, including bad breath, abdominal bloating, nausea, constipation, diarrhea, dizziness, headaches, foot pain or numbness, acid reflux (i.e., heartburn), joint or muscle pain, and muscle cramps; response options were: “not at all,” “1 day a week,” “2–3 days a week,” “4–5 days a week” and “6–7 days a week.” All participants were invited to participate in a semi-structured interview at 6 months to explore their experiences with the program and solicit feedback for improvement. Veterans were compensated with a $25 gift card for completing each study measure at 6 months and at 12 months.

We initially planned to measure participants’ weight prior to each session. However, due to the COVID-19 pandemic, we were required to deliver the program virtually and collect self-reported weight, which has previously demonstrated concordance with measured weight across diverse populations (38). Participants were instructed to self-weigh at least once per week using a home scale. A study team member called all participants approximately 1 h prior to each session to collect self-reported weight data. Participants who could not be reached prior to the session were contacted within 24 h following the session; a total of 3 attempts were made to reach each participant per session. Participants were asked to self-report weight data even when they could not attend that day’s session.

#### 2.5.1. Primary outcome measures

Primary outcome measures of feasibility and acceptability included:

1. Intervention uptake, defined in 2 ways: (a) the number of participants who enrolled in the study divided by the total number of individuals invited to participate; and (b) the number of individuals who enrolled in the study divided by the number screened by phone for eligibility.
2. Mean session attendance, defined as the number of sessions attended during the program’s core and maintenance phases divided by the number of sessions offered during each phase.
3. Study retention, defined as the number of participants who completed surveys at 6 and 12 months, respectively, divided by the number of study participants.
4. Adherence with self-weighing, defined as the number of self-reported weight measurements during the program’s core and maintenance phases divided by the total number of requested measurements.

Program acceptability was also qualitatively assessed through semi-structured interviews. Given our relatively small sample size and our desire to learn from participants’ experiences, we offered all study participants the opportunity to participate in an interview at 6 months. Interviews were conducted by a study team member with prior experience conducting qualitative interviews (CH, Project Manager). This study staff member was known to participants, as she collected pre-session weight data. Interview training consisted of mock interviews with the study’s Principal Investigator (DHG). Additionally, the first three audio recordings of participant interviews were reviewed by the Principal Investigator (DHG), and feedback was provided to the interviewer (CH) prior to conducting additional interviews. The interview guide was adapted from our prior work (23) and explored participants’ experiences with the program, including factors that supported or hindered adherence to the very low-carbohydrate meal plan. The full interview guide is shown in Appendix 4. Interviews lasted approximately 45 min in duration. Semi-structured interviews were audio recorded and transcribed verbatim. Transcripts were not returned to participants for comments or corrections.

#### 2.5.2. Secondary outcomes measures

1. Mean change in self-reported weight. Participants self-weighed at least once weekly and reported the most recent measure to a study team member prior to each session. Mean change in weight was calculated at 6 and 12 months compared to baseline.
2. Mean percent weight loss, defined as: (weight at 6 or 12 months – baseline weight) / (baseline weight) *100.
3. Achievement of ≥5% and ≥ 10% body weight loss at 6 and 12 months, defined as the percentage of participants who achieved these weight thresholds at each time point divided by the total number of participants in the analytic cohort.
4. Changes in HbA1c, LDL, HDL, and triglycerides were calculated at 6 and 12 months compared to baseline.
5. Change in survey measures, including patient-centered outcomes and self-reported physical symptoms, at 6 and 12 months compared to baseline.

### 2.6. Sample size

Consistent with guidelines and recommendations for conducting pilot studies, (39, 40) our sample size was selected to provide sufficient data regarding the feasibility and acceptability of the intervention and the methods and procedures anticipated for use in a larger scale trial (41). Specifically, we aimed to understand whether we could (1) recruit the target population, (2) retain participants in the study, (3) deliver the group-based intervention via a virtual platform, and (4) collect data at prespecified timepoints. Based on our prior pilot evaluation of the VLC-DPP among a non-Veteran population (23) and our subsequent efficacy trial informed by these pilot data (42), we considered an enrollment target of 22 total participants sufficient to provide feasibility and acceptability data. Participants were divided between 2 virtual groups, as this is the maximum number of participants typically enrolled in VA virtual weight management programs.

### 2.7. Analysis

#### 2.7.1. Quantitative analysis

Descriptive statistics were used for baseline survey response data, including demographic and characteristics and self-reported side effects. For all continuous outcomes, we calculated median change and inter-quartile ranges from baseline to 6 months and 12 months. Because mean weight change is commonly reported throughout the literature, we also calculated mean change and standard deviation in weight from baseline to 6 months and 12 months. Given our small sample and non-normal distribution of the data, we used a nonparametric statistical test, the Wilcoxon matched-pairs sign-rank test, to compare pre-post changes in survey measures and self-reported physical symptoms at 6 and 12 months compared to baseline. Further, *p*-values for each measurement difference were adjusted for multiple comparisons using the Benjamini-Hochberg procedure (43). All analyzes were conducted using R (version 4.1; R Foundation for Statistical Computing).

#### 2.7.2. Qualitative analysis

Study team members (DHG, CH) independently reviewed transcripts and collaboratively developed an interview codebook. Initial codes were developed based on interview questions and our team’s prior work (23); additional codes were added to reflect key topics that emerged from the qualitative data. Using the final codebook, the principal investigator (DHG) and a study staff member (CH) independently coded transcripts and then jointly reviewed codes to reach consensus. Minimal differences in coding were observed after 5 transcripts, and the study staff member (CH) independently coded the remaining 11 interviews. Data saturation–the point at which no new codes emerged from the data–was achieved after 12 interviews (44). Qualitative analysis was performed using NVivo software (45) and analyzed using directed content analysis (46).

#### 2.7.3. Integrated analysis

Consistent with the study’s explanatory sequential mixed method design, quantitative and qualitative data were integrated following the study period (47). Specifically, qualitative data were coded and then merged with quantitative data and themes were identified to help explain facilitators of and barriers to participants’ achievement of ≥5% weight loss.

## 3. Results

**Figure 1:**
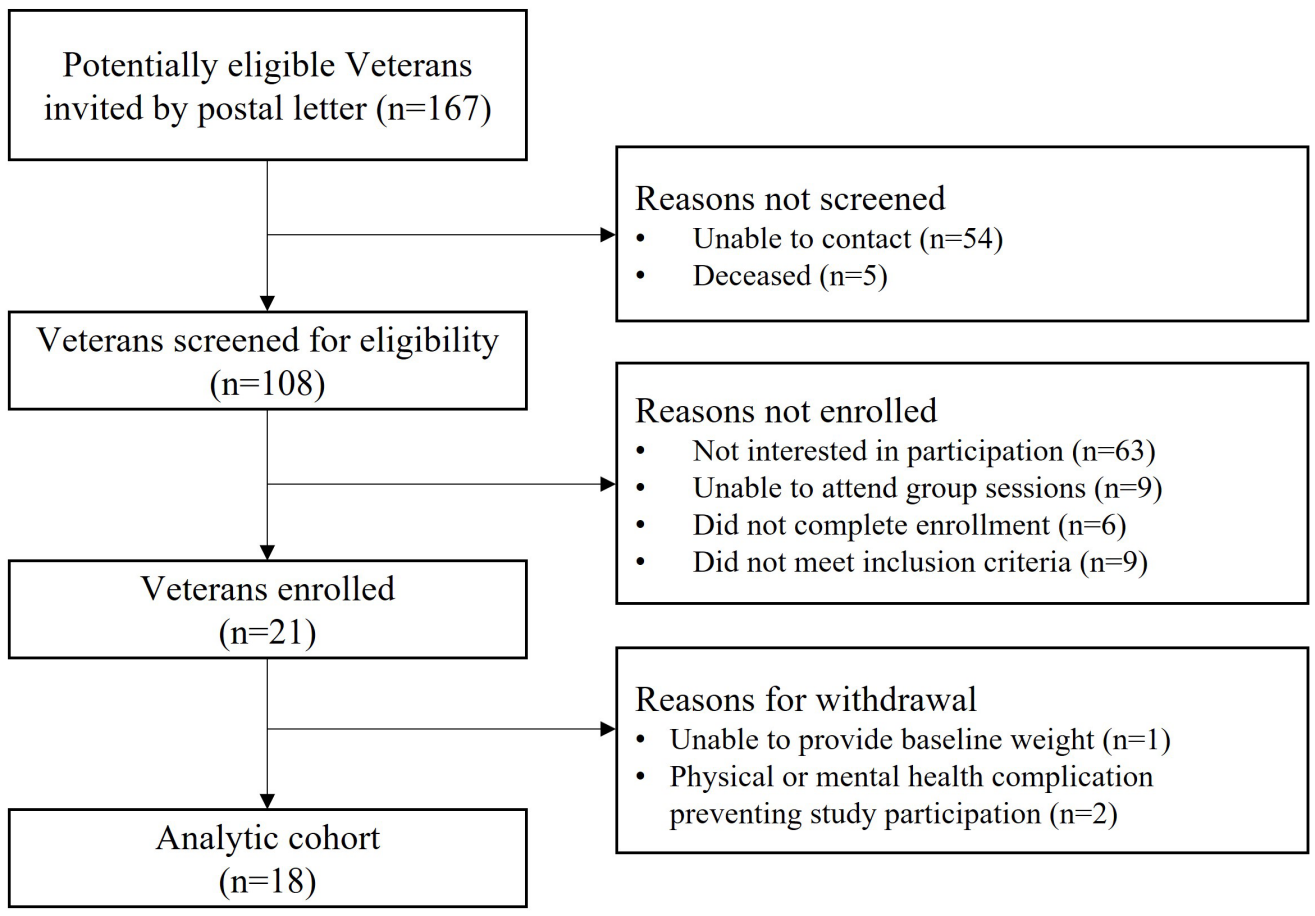
Study flow diagram.

As shown in Figure 1, 167 potentially eligible Veterans were sent a study invitation letter by postal mail. We subsequently contacted Veterans by phone and screened for eligibility until we met our recruitment target; a total of 108 Veterans were contacted. Twenty-one Veterans enrolled in the study, representing 12% (21/167) of all individuals who received a study letter and 19% (21/108) of individuals who received a study letter and were also contacted by phone. Following enrollment, 3 individuals were withdrawn from the study for reasons shown in Figure 1. Our analytic cohort consisted of 18 participants.

### 3.1. Baseline characteristics

Demographic and socioeconomic characteristics were assessed at baseline (Table 1). Thirteen (72%) participants were men, approximately half (56%) of participants were white, and the average age was 59 years. At baseline, mean BMI was 36 kg/m2 and mean HbA1c level was 6.1%. Four participants progressed from prediabetes to type 2 diabetes prior to the start of the study period.

**Table 1:** Baseline characteristics.

	All participants (*n* = 18)	Interviewed participants (*n* = 16)
Mean age in years, (SD)	59 (11)	58 (11)
Gender, *n*(%)		
Male	13 (72)	12 (75)
Female	4 (22)	3 (19)
Transgender	1 (6)	1 (6)
Race, *n* (%)		
White	10 (56)	8 (50)
Black	7 (39)	7 (44)
Multiple	1 (6)	1 (6)
Hispanic/Latino		
Non-Hispanic	17 (94)	15 (94)
Hispanic	1 (6)	1 (6)
Education > high school, *n* (%)	17 (94)	15 (94)
Married/partnered, *n* (%)	10 (56)	9 (56)
Mean weight in kg, (SD)	106.9 (18.3)	108 (19.3)
Mean BMI in kg/m^2^, (SD)	36 (5.6)	36 (5.9)
Mean % HbA1c, (SD)	6.1 (0.5)	6.1 (0.5)

BMI, body mass index; HbA1cm, hemoglobin A1c.

### 3.2. Primary outcomes

Intervention uptake is shown in Figure 1.

Mean session attendance: Participants attended a mean (SD) of 12.4 (2.9) of 16 core sessions and 3.6 (2.4) of 8 maintenance sessions. Sixteen participants attended ≥8 core sessions.

Study retention: Seventeen participants completed the 6-month survey and 15 completed the 12-month survey, resulting in 94.4 and 83.3% retention at each respective timepoint.

Adherence with self-weighing: Participants self-reported weight data for a mean (SD) of 14.5 (1.95) of 16 core sessions and 5.06 (2.37) of 8 maintenance sessions.

### 3.3. Secondary outcomes

**Figure 2:**
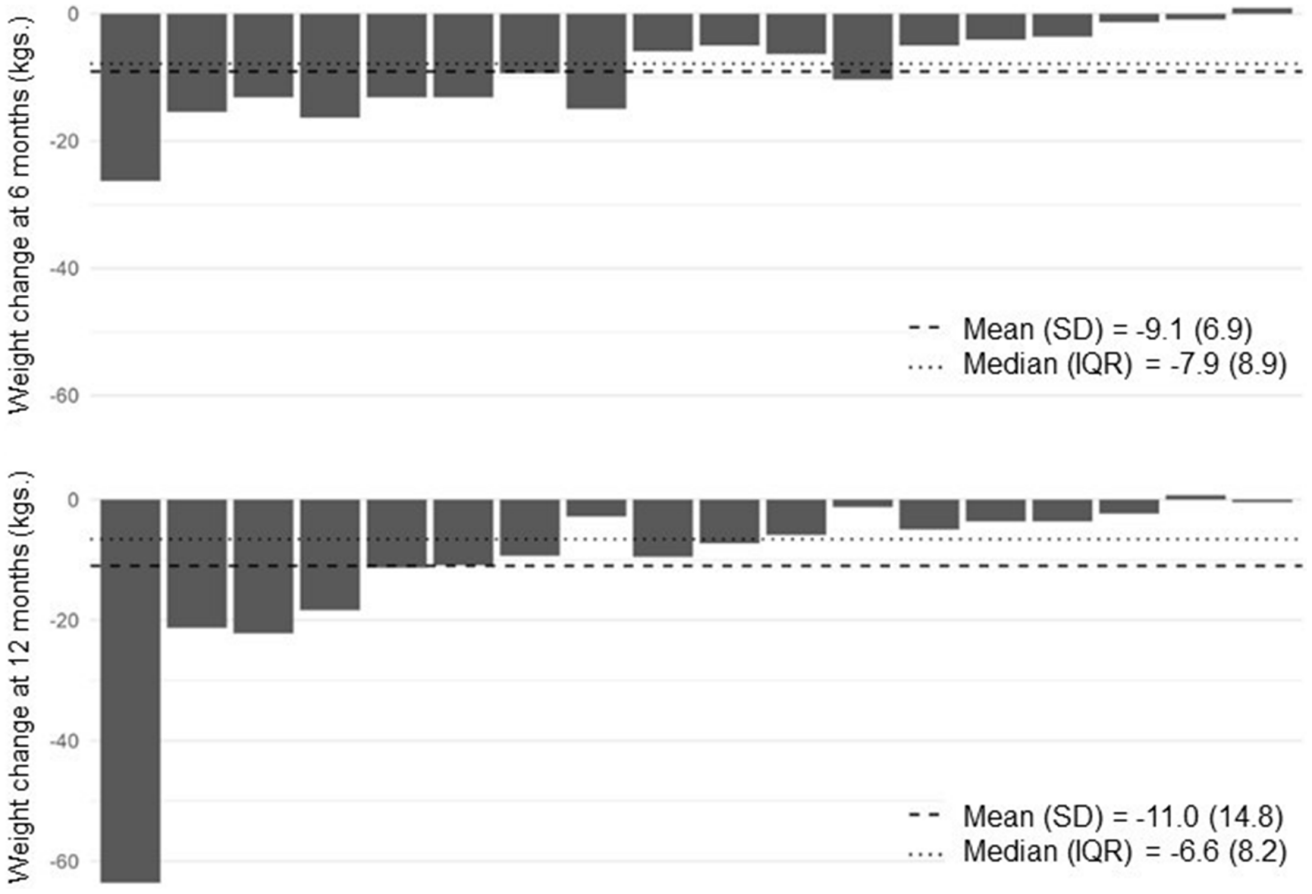
Weight change at 6 and 12 months. Each bar represents on individual study participant.

Table 2 shows weight outcomes at 6 and 12 months compared to baseline. On average, participants lost 11 kilograms at 12 months, corresponding with a mean percent weight loss of 9.45%. At 12 months, 9 participants (50%) achieved ≥5% weight loss and 7 (38.9%) achieved ≥10% weight loss. All participants with ≥5% weight loss at 12 months had achieved ≥4% weight loss by 6 months. Figure 2 shows individual participants’ weight change at 6 and 12 months compared to baseline.

**Table 2:** Weight outcomes at 6 and 12 months compared to baseline (*n* = 18).

Outcomes	Baseline	6 months	12 months
Mean weight in (kg), SD	106.9 (18.3)	97.8 (15.9)	95.9 (15.7)
Mean weight change in kg, SD	---	−9.08 (6.89)**	−11.0 (14.8)**
Median weight change in kg, IQR	---	−7.85 (−26.3, 0.907)	−6.58 (−63.6, 0.726)
Mean percent weight change	---	−8.17 (5.75)	−9.45 (10.7)
≥5% weight loss	---	11 (61.1%)	9 (50.0%)
≥10% weight loss	---	8 (44.4%)	7 (38.9%)

IQR, interquartile range.

** Denotes value of *p* < 0.001.

Table 3 shows change in HbA1c and lipids at 6 and 12 months compared to baseline. Change in HbA1c from baseline was not statistically significant at 6 months but was at 12 months (value of *p* < 0.01). Of the four participants with HbA1c ≥ 6.5% at baseline, 3 (75%) had an HbA1c <6.5% at 12-months, with two of these participants achieving ≥10% weight loss and one achieving nearly 5% weight loss. Two participants progressed from prediabetes to type 2 diabetes during the study period; one achieved <5% weight loss at 12 months and the other achieved approximately 5% weight loss at 12 months.

**Table 3:** Laboratory outcomes at 6 and 12 months compared to baseline (*n* = 18).

Outcomes (Median, IQR)	Baseline (*n* = 18)	6 months (*n* = 17)	Difference from baseline to 6 months	12 months (*n* = 17)	Difference from baseline to 12 months
HbA1c, %	6.0 (0.6)	5.7 (0.4)	−0.2 (0.5)	5.7 (0.4)	−0.3 (0.4)**
Triglycerides, mg/dL	133.5 (88.8)	108.0 (81.0)	−15.0 (76.0)	129.0 (96.0)	−10.0 (50.0)
HDL, mg/dL	41.5 (17.8)	46.0 (16.0)	2.0 (7.0)	46.0 (18.0)	2.0 (7.0)
LDL, mg/dL	107.5 (44.3)	114.0 (35.0)	3.0 (20.0)	110.0 (24.0)	3.0 (25.0)

IQR, interquartile range.

** Denotes value of *p* < 0.01.

#### 3.3.1. Change in patient-centered outcomes

Table 4 shows change in patient-centered outcomes. From baseline to 6 months, there was an improvement in patient-reported mental health (*p* < 0.001). There were no other statistically significant differences in survey measures at 6 or 12 months compared with baseline.

**Table 4:** Patient-centered outcomes at 6 and 12 months compared to baseline (*n* = 18).

Outcomes (Median, IQR)	Baseline (*n* = 18)	6 months (*n* = 17)	Difference from baseline to 6 months	12 months (*n* = 17)	Difference from baseline to 12 months
Control of Eating Questionnaire(33)^a^	51.9 (27.75)	32.5 (31.55)	−2.6 (37.75)	32.5 (19.25)	−21.3 (33.1)
Stress Eating(35)^c^	5.5 (3.0)	6.0 (2.0)	1.0 (2.5)	6.0 (1.5)	1.0 (2.0)
Palatable Eating Motives Scale(34)^b^	1.1 (1.4)	1.3 (0.9)	0.0 (0.6)	1.3 (1.0)	0.0 (0.6)
Patient-reported overall health(36)^d^	3.0 (0.75)	3.0 (1.0)	0 (1.0)	3.0 (1.0)	0 (1.0)
Patient-reported physical health(36)^e^	13.5 (5.75)	15.0 (4.0)	1.0 (2.0)	14.0 (4.5)	1.0 (3.0)
Patient-reported mental health(36)^f^	12.0 (5.0)	15.0 (5.5)	1.0 (2.5)**	14.0 (7.0)	1.0 (2.5)

IQR, interquartile range.

** Denotes value of *p* < 0.001.

a Item responses range from 0 to 100; higher value represents higher food cravings.

b 2-item scale; items are summed; score ranges from 2 to 8; higher values represent greater stress eating.

c 4-item scale; items are summed and averaged; scores range from 1 to 5; higher values represent greater stress eating.

d Single-item measure; scores range from 1 to 5; higher values represent greater overall health.

e 4-item scale; items are summed; scores range from 4 to 20; higher values represent greater patient-reported physical health.

f 4-item scale; items are summed; scores range from 4 to 20; higher values represent greater patient-reported mental health.

#### 3.3.2. Change in self-reported physical symptoms

From baseline to 6 months, there was a self-reported decrease in abdominal bloating (*p* = 0.03) and foot pain or numbness (*p* < 0.001). From baseline to 12 months, there was a self-reported decrease in bad breath (*p* = 0.03) and foot pain or numbness (*p* < 0.001). There were no other statistically significant differences in self-reported side effects at 6 or 12 months compared with baseline.

#### 3.3.3. Adverse events

There were no serious adverse events during the study period.

### 3.4. Qualitative analyzes

Sixteen participants participated in 6-month interviews. The baseline characteristics of interviewees are shown in Table 1. Mean weight change among interviewees was 8% at 6 months and 9% at 12 months and 8 (50%) achieved ≥5% weight loss at 12 months. Among interviewees who achieved ≥5% weight loss, all reported consistent adherence to the very low-carbohydrate eating pattern, which was facilitated by (a) enjoyment of low-carbohydrate foods; (b) careful monitoring of carbohydrate intake through food tracking and nutrition label reading; and (c) reduced hunger and food cravings. Representative quotes are shown in Table 5.

**Table 5:** Facilitators of weight loss among participants who achieved ≥5% weight loss at 12 months (*n* = 10).

Key theme	Representative quote (weight change at 6 and 12 months)
Enjoyment of low-carbohydrate foods	*“This program is right up my alley. I drink my water, I can eat my meat, my cheese, you know the vegetables or whatever, there’s a lot of things that, you know they say oh bologna, it’s high in fat or whatever, but I can have bologna [on this program]… [the diet] is sustainable. It’s pretty much my favorite foods.”* (Participant 14; 18% weight loss at 6 months; 43% weight loss at 12 months)
	*“I had some chicken breasts, so I sautéed them in you know lemon butter onions and hot peppers and garlic and then I sliced it real then thin and I had like chicken salad or a chicken BLT sandwich which was delicious and I can do it because I got the bread that’s one carb and then all that was pretty much zero carbs…so I went to bed a happy camper.”*(Participant 9; 14% weight loss at 6 months; 19% weight loss at 12 months)
Careful monitoring of carbohydrate intake through food tracking and nutrition label reading	*“…now whenever I shop, the first thing I look at is how many grams of carbs are in something and you know things that are supposed to be ‘low-carb,’ I take a look at the grams of carbs and I’m like, ‘are you fricking kidding me? How can you call yourself low-carb when you have 42 g of carbs per serving’…”* (Participant 8; 5% weight loss at 6 months; 5% weight loss at 12 months)
	*“You know I just came out of depression where I KNOW I fell off and put weight on and I just, I picked up my [prior food log], my meal plan that I wrote down… on how to [count carbohydrates]…it’s just so, it’s crazy! I mean I’ve tried for all these years to lose this weight and here I am, now part of this lifestyle change and man, it just fell, I’m talking about like butter melting.”* (Participant 6; 9% weight loss at 6 months; 9% weight loss at 12 months)
Reduced hunger and food cravings	*“I can say this honestly I really have not had any cravings for candy bars or sweets and stuff like that, I do not know if it’s because I’m full or because I’m more mindful of what I eat or, because I mean I stand in line at the grocery store and you are surrounded on three sides by candy bars…and I just, I do not want it, I do not want it, I do not need it, I do not care for it.”* (Participant 9; 14% weight loss at 6 months; 19% weight loss at 12 months)
	*“I’ve been on several other diets, I’ve been on another program called MOVE! at the VA and I was not successful at those programs. I think the carb as far as, the carb diet, I was able to count the carbs and still feel like I’m satisfied after I eat and when I was doing calorie counting, I was never full. So, I think that’s what made it unsuccessful for me.”* (Participant 15; 13% weight loss at 6 months; 11% weight loss at 12 months)

Among interviewees who achieved <5% weight loss at 12 months (*n* = 6), all reported factors that hindered adherence to the very low-carbohydrate eating pattern, including (a) food cravings, particularly for sweets, (b) challenges with maintaining a food log, and (c) difficulty with meal planning. Representative quotes are shown in Table 6.

**Table 6:** Barriers to weight loss among participants who achieved <5% weight loss at 12 months (*n* = 6).

Key theme	Representative quote
Food cravings	*“I’ve lost some weight and I could lose more and I’m trying to adhere to it more strictly than I have. Sometimes I have a, unfortunately I have a strong desire for sweets, I have a sweet tooth, so that makes it hard.”* (Participant 10; 4% weight loss at 6 months; 4% weight loss at 12 months)
	*“[I] have those days when you I want a bag of chips you know, I want to add fries to my meal and it seemed as though at times when I normally would not even have a craving for a candy bar, now I want a candy bar because it’s something I cannot have you know…”* (Participant 7; 1% weight loss at 6 months; 1% weight gain at 12 months)
Challenges with maintaining a food log	*“Food [logging] is still, still trying…at first I was leaving my notebook and then when I go to work or volunteer, it’s like, ‘Oh!’ so I’d write it on a piece of paper, then I say I can transfer it, sometimes I would clean out my pockets, throw away the paper, but keeping the food log is very helpful, and very effective if I remember to do it every day..”* (Participant 5; 1% weight loss at 6 months; 2% weight loss at 12 months)
	*“Trying to log everything, I really feel that there needs to be better structure built on that. I went the digital realm [using food tracking apps] and I have not learned as much as some of the people who have [logged] on paper.”* (Participant 13; 13% weight loss at 6 months; 2% weight loss at 12 months)
Difficulty with meal planning	*“…I’m not the person to plan a meal, I just, when I get home then I eat, you know I want to eat this, and I eat that, so planning the meal, you know I got kids, so getting them you know onboard, and you know changing their diet and they might not get what they usually eat..”* (Participant 7; 1% weight gain at 6 months; 1% weight loss at 12 months)
	*“I was extremely hungry, I’d taken a couple of shakes with me, the shakes were not really filling me up, I was just really ready to us to you know eat something substantial substance and I ended up running across the street to Frank Red-Hots and once I got in there, it was like, ‘Uh-oh, boy, that looks good,’ I did not eat the bun…even though I got the waffle fries and you know the BBQ sauce..”* (Participant 13; 13% weight loss at 6 months; 2% weight loss at 12 months)

Interviewees who achieved ≥5% weight loss at 12 months also discussed health benefits beyond weight loss, including a reduction in symptoms of acid reflux, increased energy, and improved glycemic control. One participant noted, *“when I first started…I had heartburn, no matter how much or how little I ate, no matter when I ate…I was living off of antacids…I thought maybe it was because I was eating so much or I was eating a particular food but it was, it had to have been the sugar because since I started doing the low-carb I have not had heartburn at all. I have four bottles now of antacids that are sitting in my medicine cabinets and in my medicine closet and I do not even use them anymore* (Participant 8)*.”* Another participant noted, *“I feel that this program has given me a new lease on life. I’m a lot more positive, I have a lot more energy, I feel better about myself, I have, you know all the way around I think I’m a better person because of this program* (Participant 15)*.”*

Regardless of weight change, participants acknowledged challenges with navigating the high-carbohydrate food environment, particularly when *“you cannot control the meal that’s being served…if you get out on the run and have to get something to eat or if you are invited as a guest to an event or someone’s house…*(Participant 17).*”* Two interviewees noted higher food costs when following a low-carbohydrate meal plan but did not feel that this was a barrier to dietary adherence. Three interview participants reported stable food costs and three reported a reduction in food costs. One interview participant noted a higher cost of individual low-carbohydrate food items but a reduction in total spending on food, *“because I’m eating less. By choosing healthier low-carb options such as vegetables, meats things like that, I’m staying fuller, I feel like I’m staying fuller longer off one meal* versus *when I was eating processed snacks and you know fast food and quick food items…I’m not buying so much junk food that you eat it and then you are hungry, you know a couple minutes later* (Participant 17)*.”*

## 4. Discussion

This pilot study tested the feasibility and acceptability of a virtual very low-carbohydrate Diabetes Prevention Program among Veterans with overweight or obesity and prediabetes. Approximately 12% of Veterans who received an invitation letter enrolled in the study, and once enrolled, participants demonstrated high rates of mean session attendance (12 out of 16 core sessions) with 76% of participants attending ≥8 core sessions. VLC-DPP uptake was similar to MOVE! uptake (10) but lower than uptake in an online version of the CDC’s standard DPP in which nearly one-quarter of invited Veterans enrolled in the program (48). In contrast, VLC-DPP participants’ engagement was comparatively higher than engagement in MOVE! or standard DPP. A systematic review of MOVE! studies showed a maximum of 25% of MOVE! participants completing 5 or more sessions within 6 months, (11) and a clinical demonstration trial comparing VA MOVE! to the CDC’s NDPP showed only 31% of Veterans completed 8 MOVE! sessions in 6 months (vs. 42.5% of DPP participants) (8). Factors contributing to Veterans’ high rate of VLC-DPP engagement may include the program’s virtual format, its emphasis on carbohydrate rather than fat and calorie restriction, and its weight loss effectiveness.

Participants achieved a mean percent weight loss of 8.17% at 6 months and, on average, continued to lose weight during the study period, achieving a mean percent weight loss of 9.45% at 12 months. While study participants achieved greater mean weight loss than non-Veteran participants in our prior pilot work (5.2% at 12 months) (23), both cohorts demonstrated continued mean weight loss during the 12-month study period (23). Mean weight loss was similar in magnitude to other very low-carbohydrate dietary interventions (49, 50), and corresponded to achievement of ≥5% weight loss among half of study participants. In contrast, a systematic review of MOVE! program outcomes showed modest weight loss at 12 months (+0.13 kg to −3.3 kg at 12 months), with only 19 to 25% of participants achieving ≥5%. Prior work testing the comparative weight loss effectiveness of MOVE! with the standard DPP similarly demonstrated modest weight loss at 12 months without significant between group differences (−2.0 kg MOVE! vs. -3.4 kg DPP) (8). Among Veteran participants in an online DPP, mean weight loss at 12 months was−4.0 kg, corresponding to 3.7% body weight loss.

The effect of any specific diet on weight loss varies markedly between individuals, (14) and our data predictably shows wide variation in individual participants’ weight change, with nearly 40% achieving ≥10% weight loss and 50% achieving <5% weight loss. Numerous studies have aimed to identify pretreatment psychological predictors of weight loss but have failed to show consistent findings (51, 52). In contrast, early weight loss achievement (e.g., within 6 months) is a reliable predictor of future weight loss and weight maintenance (53–58). Little is known about the factors that lead to early weight loss, though a reduction in hunger and food cravings compared to baseline levels may be a key contributor (59). In this study, all participants who achieved ≥5% weight loss at 12 months had achieved ≥4% weight loss by 6 months. Qualitative interview data suggests that Veterans’ enjoyment of low-carbohydrate foods, reduced levels of hunger and food cravings when following a low-carbohydrate eating pattern, and perceived health benefits, including weight loss, are key contributors to program’s acceptability and weight loss effectiveness. These findings are consistent with our prior pilot work among a non-Veteran population (23), and suggest that early assessment of participants’ enjoyment of the meal plan and levels of hunger and cravings may help to identify individuals likely to achieve ≥5% weight loss with carbohydrate-restriction and those who may need a different intervention (e.g., alternative diet) or additional support (e.g., anti-obesity medication) to achieve this goal.

## 5. Limitations

This study had several limitations. First, we recruited individuals from a single VA healthcare system so the results may not be generalizable to other VA sites or non-Veteran populations. Second, we did not evaluate outcomes beyond 12 months, and we are therefore unable to assess long-term adherence to a carbohydrate-restricted meal plan. However, growing data suggests that dietary carbohydrate restriction can be a sustainable eating pattern (22, 60–62). Third, although our qualitative data provide some insight into the association between participants’ dietary adherence and weight change, we did not formally assess dietary adherence using validated measures. Consistent with other lifestyle change programs such as the VA’s MOVE! Weight Management Program and the CDC’s NDPP, we considered weight change to be a surrogate measure of dietary adherence in this pilot study. Fourth, due to the COVID-19 pandemic and transition to a virtual intervention, we collected self-reported weight data, which may not reflect participants’ actual weight, though prior literature suggests concordance between self-reported and measured weight (38). Finally, because this was a single-arm pilot study, we were not able to compare the intervention’s weight loss clinical effectiveness with other lifestyle change programs such as MOVE! or the NDPP.

## 6. Conclusion

Our pilot findings demonstrate the feasibility, acceptability, and preliminary efficacy of a very low-carbohydrate dietary change program among Veterans with overweight and prediabetes. The VA MOVE! Weight Management Program provides an essential foundation for VA efforts to help Veterans lose weight and improve cardiometabolic health through lifestyle change focused on calorie and dietary fat restriction and increased physical activity. However, as “one-size-fits-all” dietary approaches may not meet individuals’ diverse preferences and needs (14), it is necessary to incorporate other dietary change approaches into clinical practice. Accordingly, the VA/DoD Clinical Practice Guideline for the Management of Adult Overweight and Obesity recommends the use of varied dietary change approaches including low- and very low-carbohydrate diets to expand the menu of preference-sensitive treatment options (12). Our team is currently testing the weight loss and glycemic effectiveness of the VLC-DPP compared to the CDC’s standard NDPP in a fully powered randomized controlled trial (42, 63). These data may inform future efforts to implement, evaluate, and scale the VLC-DPP within VHA as one alternative to the standard MOVE! curriculum for patients with prediabetes or risk factors for T2DM.

## Data availability statement

The deidentified datasets analyzed for this study may be requested from the study team and access may be provided pending review and approval by the VA IRB. Requests to access the datasets should be directed to dhafez@med.umich.edu.

## Ethics statement

This study involving human participants was approved by the VA Ann Arbor Healthcare System (VAAAHS) Institutional Review Board. The patients/participants provided their written informed consent to participate in this study.

## Author contributions

LS, CR, DG, JK, and MH contributed to the design of the intervention. CH led study recruitment, quantitative data collection, and qualitative data collection and analysis. JM led the intervention. RE conducted quantitative data analyzes. DG drafted the manuscript, which was reviewed, edited, and approved by all co-authors. All authors contributed to the article and approved the submitted version.

## Funding

This research was supported by the US Department of Veterans Affairs Health Services Research and Development (LIP 20–122) through the Ann Arbor Center for Clinical Management Research (CCMR). Additional support was provided by the National Institute of Diabetes and Digestive and Kidney Diseases (Grant Number P30DK092926).

## Conflict of interest

DG received consulting fees from the National Kidney Foundation of Michigan. JK has received consulting fees from SeeChange Health, HealthMine, the Kaiser Permanente Washington Health Research Institute, and the Washington State Office of the Attorney General; and honoraria from the Robert Wood Johnson Foundation, AbilTo, Inc., the Kansas City Area Life Sciences Institute, the American Diabetes Association, the Luxembourg National Research Fund, and the Donaghue Foundation. LS received consulting fees from Sentinel Management, LLC.

The remaining authors declare that the research was conducted in the absence of any commercial or financial relationships that could be construed as a potential conflict of interest.

## Publisher’s note

All claims expressed in this article are solely those of the authors and do not necessarily represent those of their affiliated organizations, or those of the publisher, the editors and the reviewers. Any product that may be evaluated in this article, or claim that may be made by its manufacturer, is not guaranteed or endorsed by the publisher.
