# Supplementary material for: A very low-carbohydrate diabetes prevention program for veterans with prediabetes: a single-arm mixed methods pilot study
Source: Front Nutr. 2023 May 17;10:1069266. doi: 10.3389/fnut.2023.1069266 (PMC10230095; doi:10.3389/fnut.2023.1069266)
Supplement: Supplementary file 1 [file Data_Sheet_1.docx]

**Appendix 1.** Template for Intervention Description and Replication (TIDieR) checklist (29).

| **TIDieR checklist Item** | **Item Description** | **Very Low-Carbohydrate Diabetes Prevention Program Intervention Description** |
| --- | --- | --- |
| 1 – Name | Provide the name or a phrase that describes the intervention. | Very Low-Carbohydrate Diabetes Prevention Program (VLC-DPP) for Veterans |
| 2 – Why | Describe any rationale, theory, or goal of the elements essential to the intervention. | Veterans who receive care within the Veterans Health Administration (VHA) are disproportionately burdened by obesity and its cardiometabolic consequences compared to non-Veteran populations. Current resources and programs provide an essential foundation for weight management in the VHA but most participants do not achieve clinically significant weight loss. Effective strategies that build on this foundation are needed to further engage and support weight loss among Veterans with obesity.  The VA/Department of Defense (DoD) Clinical Practice Guideline (CPG) for the Management of Adult Overweight and Obesity recommends the use of preference-sensitive dietary change approaches, as the effect of any specific diet on weight loss varies markedly between individuals and diet choice may play a key role in engaging individuals in weight management treatment. Low-carbohydrate diets consisting of foods such as meat, poultry, fish, eggs, tofu, tempeh, nuts, seeds, leafy greens, and other non-starchy vegetables are one evidence-based dietary approach with known appeal among Veterans. Low- and very low-carbohydrate diets—commonly defined as 10-26% and < 10% total daily energy from carbohydrate, respectively—can support weight loss, glycemic control, and favorable changes in cholesterol, blood pressure, and self-reported measures of energy, hunger, and food cravings while reducing the need for medications to control chronic conditions. Accordingly, a low-carbohydrate lifestyle change program may be one promising strategy to expand the menu of evidence-based weight management treatment options for Veterans with overweight and obesity. |
| 3 – What Materials | Describe any physical or informational materials used in the intervention, including those provided to participants or used in intervention delivery or in training of intervention providers. | Participants attended 16 weekly sessions delivered over a 6-month period (core phase) and 7 bimonthly/monthly sessions for the remaining 6 months (maintenance phase). Sessions were delivered virtually and in groups by a Registered Dietician (RD). Each session included written information that was presented by the RD to the group using the screen share feature of the virtual platform. The same written information was printed and provided to participants. Sessions encouraged participants to transition to a very low-carbohydrate diet, to increase their physical activity, and to otherwise improve their health and well-being. Session topics are listed in **Appendix 2**. Participants were also directed to relevant online resources for each session.  The RD had a coaching guide to to aid in the delivery of each session; the guide included the session’s instructional content as well as questions and prompts to facilitate group-based discussion.  Participants who did not have a webcam and/or bodyweight scale were provided necessary materials by the study team. |
| 4 – What Procedures | Describe each of the procedures, activities, and/or processes used in the intervention, including any enabling or support activities. | Delivery: All sessions were delivered via closed-group video calls (i.e., the same participants are present in each session). Groups contain up to 10 participants. Sessions were 60 minutes in duration. All participants received educational information in each session with time for questions and discussion.  Tracking: Participants were asked to self-weigh at least once per week using a home scale. Participants were asked to track dietary carbohydrate intake using a paper log or an app such as MyFitnessPal or Carb Manager. Participants were also asked to track minutes of physical activity per week.  Diet: Participants were taught to slowly transition to a very low-carbohydrate diet (20-35 grams of net carbohydrates per day). The diet includes no calorie limit or specific meal plans. Participants were encouraged to eat when hungry and stop when satisfied and to explore a wide variety of low-carbohydrate foods that fit into their food budget and preferences. After 6 months, if participants had met their weight loss goal and/or desired to increase their carbohydrate intake, they were instructed to gradually increase their carbohydrate intake (i.e., add 5 grams of net carbohydrates per week) and observe changes in body weight, hunger, or food cravings. They were instructed to reduce carbohydrate intake if they noticed increases in weight, hunger, or food cravings.  Physical activity: Participants were encouraged to engage in at least 150 minutes of moderate intensity physical activity per week. |
| 5 – Who Provided | For each category of intervention provider (such as psychologist, nursing assistant), describe their expertise, background, and any specific training given. | All sessions were led by a VA RD trained in diabetes education and low-carbohydrate nutrition counseling using the intervention materials and online resources ([CME course - Diet Doctor](https://www.dietdoctor.com/cme)) The principal investigator, a primary care provider with training in obesity medicine and experience with low carbohydrate nutrition counseling, provided additional training and attended all sessions to assist with participant questions. |
| 6 – How | Describe the modes of delivery (such as face to face or by some other mechanism, such as internet or telephone) of the intervention and whether it was provided individually or in a group. | All sessions were held virtually using the VA Video Connect platform. Participants connected on their own devices (e.g., smartphones, laptops, etc.) with an internet connection. Groups contained up to 10 participants and one facilitator (RD). The principal investigator and study project manager were also present at all virtual sessions.  A study team member called all participants approximately one hour prior to each session to collect self-reported weight data. Participants who could not be reached prior to the session were contacted within 24 hours following the session; a total of 3 attempts were made to reach each participant per session. Participants were asked to self-report weight data even when they could not attend that day’s session. |
| 7 – Where | Describe the type(s) of location(s) where the intervention occurred, including any necessary infrastructure or relevant features. | The intervention was conducted among individuals who received primary care within the VA Ann Arbor Healthcare System (VAAAHS), which includes the Lieutenant Colonel Charles S. Kettles VA Medical Center (VAMC) and six other freestanding VA outpatient health clinics serving a diverse patient population throughout southeast Michigan and Toledo, Ohio. |
| 8 – When and How Much | Describe the number of times the intervention was delivered and over what period of time including the number of sessions, their schedule, and their duration, intensity, or dose. | Sessions were held weekly for the first 6 months (16 sessions, with weeks off for holidays, etc.). The remaining 7 sessions were held approximately monthly for the last 6 months of the intervention. All 23 sessions were 1 hour and held on a consistent schedule (either Monday afternoons or Wednesday mornings depending on participant preference at baseline). |
| 9 - Tailoring | If the intervention was planned to be personalized, titrated or adapted, then describe what, why, when, and how. | Participants were encouraged to explore a variety of low-carbohydrate foods that appealed to their preferences and budget constraints. Participants were not provided with specific meal plans but rather provided with a variety of low-carbohydrate alternatives to high-carbohydrate foods and meals. For example, low-carbohydrate breakfast options included scrambled eggs, an omelet with broccoli and cheese, full-fat unsweetened Greek yogurt with pecans, a crustless quiche, low-carbohydrate pancakes, waffles or muffins, or a low-carbohydrate shake. Participants were taught to search for recipes online using search teams like “low-carb pancakes” and reviewing nutrition facts to select options with approximately 5 grams of net carbohydrates per serving. Participants were encouraged to share their favorite low-carbohydrate foods and meals with classmates during group sessions.  During the maintenance phase, participants were advised that they could maintain their very low-carbohydrate eating pattern or they could gradually liberalize their daily carbohydrate intake. Participants desiring to add carbohydrates to their eating plan were advised to increase their daily carbohydrate intake by 5 grams of net carbohydrates and maintain that change for at least one week. Participants were advised to continue to self-weigh and to use increases in body weight, hunger, or food cravings as an indication that the carbohydrate intake was too high. In contrast, weight stability or continued weight loss without excessive hunger or cravings would indicate a well-tolerated carbohydrate level. |
| 10 – Modifications | If the intervention was modified during the course of the study, describe the changes. | The intervention was intended to be delivered through in-person, group-based sessions. However, due to the COVID-19 pandemic, the intervention was delivered remotely. |
| 11 – How Well (planned) | If intervention adherence or fidelity was assessed, describe how and by whom, and if any strategies were used to maintain or improve fidelity, describe them. | The principal investigator attended all sessions to assist to ensure the information was delivered as intended; there were no formal measures of fidelity used during this pilot study. |
| 12 – How Well (actual) | If intervention adherence or fidelity was assessed, describe the extent to which the intervention was delivered as planned. | The principal investigator attended all sessions to assist to ensure the information was delivered as intended; there were no formal measures of fidelity used during this pilot study. |
