# Supplementary material for: A very low-carbohydrate diabetes prevention program for veterans with prediabetes: a single-arm mixed methods pilot study
Source: Front Nutr. 2023 May 17;10:1069266. doi: 10.3389/fnut.2023.1069266 (PMC10230095; doi:10.3389/fnut.2023.1069266)
Supplement: Supplementary file 2 [file Data_Sheet_2.docx]

**Appendix 2.** Session topics for the Center for Disease Control and Prevention’s (CDC’s) National Diabetes Prevention Program (NDPP) and session topics for the Very Low-Carbohydrate Diabetes Prevention Program (VLC-DPP). VLC-DPP sessions with substantial modifications to the dietary content are denoted with an asterisk (*). Physical activity and behavior change sessions were minimally modified from the original CDC NDPP version. Minimal modifications included (1) strategies for being physically active when following a very low-carbohydrate meal plan and (20 replacing references to low-calorie, low-fat foods with very low-carbohydrate alternatives; these sessions are denoted with two asterisks (**).

| **Session** | **CDC’s NDPP Session Topic** | **VLC-DPP Session Topic** |
| --- | --- | --- |
| **Core Phase** | | |
| 1 | Welcome to the National Diabetes Prevention Program | Welcome to the LC-DPP** |
| 2 | Be a Fat and Calorie Detective | Be a Carbohydrate Detective* |
| 3 | Three Ways to Eat Less Fat and Fewer Calories | Low-Carb Meals* |
| 4 | Health Eating | Low-Carb Cooking and Shopping* |
| 5 | Move those Muscles | Move those Muscles** |
| 6 | Being Active – A Way of Life | Being Active – A Way of Life** |
| 7 | Tip the Calorie Balance | Challenges and Support** |
| 8 | Take Charge of What’s Around You | Take Charge of What’s Around You** |
| 9 | Problem Solving | Problem Solving** |
| 10 | Four Keys to Healthy Eating Out | Four Keys to Low-Carb Eating Out* |
| 11 | Talk Back to Negative Thoughts | Talk Back to Negative Thoughts** |
| 12 | The Slippery Slope of Lifestyle Change | The Slippery Slope of Lifestyle Change** |
| 13 | Jump Start Your Activity Plan | Jump Start Your Activity Plan** |
| 14 | Make Social Cues Work for You | Make Social Cues Work for You** |
| 15 | You Can Manage Stress | You Can Manage Stress** |
| 16 | Ways to Stay Motivated | Ways to Stay Motivated** |
| **Maintenance Phase** | | |
| 17 | Welcome to Months 7-12 | Welcome to Months 7-12* |
| 18 | Handling Holidays, Vacations, and Special Events | Handling Holidays, Vacations, and Special Events* |
| 19 | Fats – Saturated, Unsaturated, and Trans Fat | Fats – Saturated, Unsaturated, and Trans Fat* |
| 20 | Staying on Top of Physical Activity | Staying on Top of Physical Activity** |
| 21 | Healthy Eating with Variety and Balance | Revisiting Recipes and Cooking* |
| 22 | Stress and Time Management | Stress and Time Management** |
| 23 | Long-term Maintenance and Looking Forward | Long-term Maintenance and Looking Forward** |
