# Supplementary material for: A very low-carbohydrate diabetes prevention program for veterans with prediabetes: a single-arm mixed methods pilot study
Source: Front Nutr. 2023 May 17;10:1069266. doi: 10.3389/fnut.2023.1069266 (PMC10230095; doi:10.3389/fnut.2023.1069266)
Supplement: Supplementary file 3 [file Data_Sheet_3.docx]

| **Appendix 3.** Summary of survey items, response options, and scoring. The question item numbers listed in the second column correspond to the item number in the original survey instrument. | | | |
| --- | --- | --- | --- |
| **Control of Eating Questionnaire (CoEQ)**  Dalton, M., Finlayson, G., Hill, A., Blundell, J. Preliminary validation and principal components analysis of the Control of Eating Questionnaire (CoEQ) for the experience of food craving. (2015). *European Journal of Clinical Nutrition, 69,* 1313-1317.  *Note: The CoEQ has 21 questions across 6 sections; we include 2 sections in our survey to measure carvings for sweet and savory foods.* | | | |
| *Over the past 7 days…* | | | |
| How strong was your desire to eat sweet foods? | Q3 | Answer choices on a sliding scale from 0 to 100 with anchors at 0 and 100: 0- not at all strong 10 20 30 40 50 60 70 80 90 100- extremely strong | Items are summed and averaged. |
| How strong was your desire for non-sweet foods like French fries, potato chips, hamburgers, pizza? | Q3 (adapted) |  |  |
| *Over the past 7 days, how often have you had cravings for the following:* | | | |
| Sweet foods like candy, chocolate, and ice cream? | Q13 | Answer choices on a sliding scale from 0 to 100 with anchors at 0 and 100:  0- not at all  10  20  30  40  50  60  70  80  90  100- extremely often | Items are summed and averaged. |
| Other sweet foods (cakes, pastries, cookies, etc.)? | Q14 |  |  |
| Fruit or fruit juice? | Q15 |  |  |
| Dairy foods (cheese, yogurts, milk, etc.)? | Q16 |  |  |
| Starchy foods (bread, rice, pasta, etc.)? | Q17 |  |  |
| Foods that are not sweet like French fries, potato chips, burgers, pizza, etc.? | Q18 |  |  |
| **2-item measure of stress eating**  Tsenkova, V., Boylan, J. M., & Ryff, C. (2013). Stress eating and health. Findings from MIDUS, a national study of US adults. *Appetite, 69*, 151-155. | | | |
| *Please indicate how you usually experience a stressful event:* | | | |
| I eat more of my favorite foods to make myself feel better. | Q1 | Answer choices 1 through 4 with anchors on 1 and 4:  1- Not at all  2  3  4- A lot | Items are summed. Higher scores indicate greater use of food in response to stress. |
| I eat more than I usually do. | Q2 |  |  |
| **Palatable Eating Motives Questionnaire – Coping Subscale**  Burgess, E. E., Turan, B., Lokken, K. L., Morse, A., & Boggiano, M. M. (2014). Profiling motives behind hedonic eating. Preliminary validation of the Palatable Eating Motives Scale. *Appetite, 72*, 66-72. | | | |
| *Below is a list of reasons that people sometimes give for eating tasty foods and drinks such as:*  *- Sweets like chocolate, doughnuts, cookies, cake, candy, ice cream, other desserts.*  *- Salty snacks like chips, pretzels, and crackers.*  *- Fast foods like hamburgers, cheeseburgers, pizza, fried chicken and French fries.*  *- Sugary drinks like soda, sweet tea, milkshakes, and sweet coffee drinks.*  *Thinking of all the times you ate these kinds of foods/drinks, how often would you say that you ate/drank for the following reasons? Choose the answer that best describes you.* | | | |
| To forget your worries. | Q1 | 1 = Almost never/Never  2 = Some of the time  3 = Half of the time  4 = Most of the time  5 = Almost always/Always | Items are summed and averaged. |
| Because it helps you when you feel depressed or nervous. | Q4 |  |  |
| To cheer up when you are in a bad mood. | Q6 |  |  |
| To forget about your problems. | Q17 |  |  |
| **Patient-Report Outcomes Measurement Information System (PROMIS) – Global Health**  Cella, D., Riley, W., Stone, A. A., Rothrock, N., Reeve, B. B., Yount, S., Amtmann, D., D., B., Choi, S., Cook, K. F., et al. (2010). The Patient Reported Outcomes Measurement Information System (PROMIS) developed and tested its first wave of adult self-reported health outcome item banks: 2005-2008. *Journal of Clinical Epidemiology 63*, 1179-1194. | | | |
| Please respond to each item by marking one box per row. | | | |
| In general, would you say your health is: | Global01 | 5- Excellent  4- Very good  3- Good  2- Fair  1- Poor | Global Physical Health raw score, sum of: Global03, Global06, Global07rc, Global08r  Global Mental Health raw score, sum of: Global02, Global04, Global05, Global10r  Raw sum to T-score tables in Appendix 1 tables, raw response scores from remaining two items used in analyses  Scoring for Q10/Global07rc:  (response) 0 = 5 (score)  1 = 4  2 = 4  3 = 4  4 = 3  5 = 3  6 = 3  7 = 2  8 = 2  9 = 2  10 = 1  The interpretation of summed scores can be found in the table in the following citation:  Hays, R. D., Bjorner, H., Revicki, R. A., Spritzer, K. L., & Cella, D. (2009). Development of physical and mental health summary scores from the Patient Reported Outcomes Measurement Information System (PROMIS) global items. Quality of Life Research, 187), 873-80. (PMCID: PMC2724630) |
| In general, would you say your quality of life is: | Global02 |  |  |
| In general, how would you rate your physical health? | Global03 |  |  |
| In general, how would you rate your mental health, including your mood and your ability to think? | Global04 |  |  |
| In general, how would you rate your satisfaction with your social activities and relationships? | Global05 |  |  |
| In general, please rate how well you carry out your usual social activities and roles. (This includes activities at home, at work and in your community, and responsibilities as a parent, child, spouse, employee, friend, etc.) | Global09r |  |  |
| To what extent are you able to carry out your everyday physical activities such as walking, climbing stairs, carrying groceries, or moving a chair? | Global06 | 5- Completely  4- Mostly  3- Moderately  2- A little  1- Not at all |  |
| *In the past 7 days:* | | |  |
| How often have you been bothered by emotional problems such as feeling anxious, depressed, or irritable? | Global10r | 5- Never  4- Rarely  3- Sometimes  2- Often  1- Always |  |
| How would you rate your fatigue on average? | Global08r | 5- None  4- Mild  3- Moderate  2- Severe  1- Very severe |  |
| How would you rate your pain on average? | Global07rc | Answer choices 0 through 10 with anchors on 0, 10:  0- No pain  1  2  3  4  5  6  7  8  9  10- Worst pain imaginable |  |
| **Treatment Self-Regulation Questionnaire**  Levesque, C., Williams, G., Elliot, D., Pickering, M., Bodenhamer, B., Finley, P. (2007). Validating the theoretical structure of the Treatment Self-Regulation Questionnaire (TSRQ) across three different health behaviors. *Health Education Research, 22(5)*, 691-702.  Full scale available at: <https://selfdeterminationtheory.org/health-care-self-determination-theory-questionnaire/> | | | |
| *There are a variety of reasons why patients may take steps to prevent diabetes. Please consider the following behaviors and indicate how true each of these reasons is for you. The reason I would take steps to prevent diabetes is:* | | | |
| Because I feel that I want to take responsibility for my own health. | Q1 | Answer options 1-7 with anchors given at 1,4,7:  1- Not true at all  2  3  4- Somewhat true  5  6  7- Very true | Autonomous score:  Summed and averaged responses for items 1, 3, 6, 8, 11, 13.  Controlled score:  Summed and average responses for items: 2, 4, 7, 9, 12, 14.  Amotivation score:  Summed and average responses for items 5, 10, 15. |
| Because I would feel guilty or ashamed of myself if I didn't take steps to prevent diabetes. | Q2 |  |  |
| Because I personally believe it is the best thing for my health. | Q3 |  |  |
| Because others would be upset with me if I didn't take steps to prevent diabetes. | Q4 |  |  |
| I really don't think about it. | Q5 |  |  |
| Because I have carefully thought about it and believe it is very important for many aspects of my life. | Q6 |  |  |
| Because I would feel bad about myself if I didn't take steps to prevent diabetes. | Q7 |  |  |
| Because it is an important choice I really want to make. | Q8 |  |  |
| Because I feel pressure from others to take steps to prevent diabetes. | Q9 |  |  |
| Because it is easier to do what I am told than think about it. | Q10 |  |  |
| Because it is consistent with my life goals. | Q11 |  |  |
| Because I want others to approve of me. | Q12 |  |  |
| Because it is very important for being as healthy as possible. | Q13 |  |  |
| Because I want others to see I can do it. | Q14 |  |  |
| I don't really know why. | Q15 |  |  |
