# Supplementary material for: A very low-carbohydrate diabetes prevention program for veterans with prediabetes: a single-arm mixed methods pilot study
Source: Front Nutr. 2023 May 17;10:1069266. doi: 10.3389/fnut.2023.1069266 (PMC10230095; doi:10.3389/fnut.2023.1069266)
Supplement: Supplementary file 4 [file Data_Sheet_4.docx]

**Appendix 4.** Low-Carbohydrate Diabetes Prevention Program interview guide

**Introduce self**

Hello, my name is [name of interviewer].

**Discuss purpose of the interview group**

We are interested in understanding your experiences to date with the Low-Carbohydrate Diabetes Prevention Program. We want to understand how the program has changed your diet and physical activity habits, and we want to know your thoughts about what you liked about the program and ways that it could be improved.

I want to encourage you to answer honestly and share any thoughts you may have. There is no right or wrong answer, and you will not be penalized for anything you say. We care about your opinion, so that we can make an even better program that could benefit others.

You will receive a $25 gift card for participating in this interview.

**Describe how we will assure confidentiality and answer any questions.**

I want to take a minute to tell you what happens with the information you provide for us today. Your participation is voluntary and will in no way affect the care you receive here at the VA. You may stop at any time and you do not have to answer any question you do not wish to answer. We’d like to audio-record your responses to the questions asked during this interview so that we accurately capture everything you say. I want to assure you that the information you provide will remain confidential. We will assign a study number to the notes pertaining to the phone call and everything is stored on a secure server where only authorized study staff have access. Is that okay for you?

As a reminder, you are not obligated to answer any question you feel uncomfortable responding to, and you are not required to participate. You may leave the interview at any time.

Do you have any questions for me before we begin?

- **If Yes: (Turn on recorder)** Thank you, I have turned on the recorder. The remainder of our conversation will be recorded. I just want to confirm, is it okay with you that this is recorded for research purposes?
- **If No:** No problem. I will be taking detailed notes, so that I can capture your responses. If I pause at any time, just know it may be just because I’m jotting down notes. I may ask you a few follow up questions, just to make sure I’m capturing everything.

**Interview**

General experience

1. Tell me about how the program is going for you so far.
   1. Tell me more.
   2. Probe, if needed: what do you like about the program?
      1. Tell me more.
   3. Probe, if needed: what, if anything, has been hard or challenging about the program?
   4. Probe, if needed: Any side effects?
   5. Tell me more.

Due to COVID we had to conduct the group online using the virtual care platform. I want to ask you some questions specific to using the virtual care platform.

1. Tell me about how it was to use the virtual care platform for this study?
2. This study required you to come to the VA very minimally (for lab work). Tell me about how it was to participate in a group that met virtually?
   1. Probe, if needed: did you encounter any barriers?
   2. Probe, if needed: did you experience anything positive?
3. Tell me if you had a reaction/thought about completing a study conducted remotely/online?
   1. Probe, if needed: would you have been interested in participating in the study if it required you to come to the VA and met in person?
      1. Probe, if needed: did you encounter any barriers?
      2. Probe, if needed: did you experience anything positive?
4. Tell me about what difficulties, if any, did you face in logging onto the group?
   1. Did you face any technical difficulties? Can you tell me more about that?

Diet advice and experience

1. To what extent did you consider the carbohydrate content of your food before starting this program, if at all?
   1. Probe, How familiar were you with what foods included carbohydrates?
2. Tell me specifically about how the low-carbohydrate diet is going for you.
   1. Tell me more.
   2. Probe, if needed: has anything surprised you about this meal plan?
   3. Probe, if needed: what, if anything, has been challenging about sticking to this meal plan?
3. Tell me about how your dietary habits and routines have changed with this program, if at all.
   1. Tell me more.
4. Tell me about how the low-carb diet influenced your food spending, if at all?
   1. Is cost a barrier to continuing to follow this diet over time?
5. Tell me about how it was to find low-carb foods when grocery shopping?

Physical activity advice and experience

1. Tell me about how your physical activity habits and routines have changed with this program, if at all.
   1. Tell me more.
   2. Probe, if needed: what, if anything, has been challenging about getting 30 minutes of physical activity, 5 days per week?
2. Tell me your thoughts on how effective this program may be without the exercise component?
   1. Would you have preferred a program that focused only on diet?

Motivation and expectations

1. Tell me about your motivation for joining the program?
   1. Tell me more.
   2. Probe, if needed: What made you decide to sign up for this program?
2. Before participating in this program, what other ways had you tried to lose weight or to prevent diabetes?
   1. If participant previously took part in traditional DPP:
      1. Tell me about how your experience with this low-carbohydrate DPP compares to your prior experience with the traditional low-fat DPP.
3. In what ways has this program met or not met your expectations?
   1. Tell me more.
   2. Probe, if needed: has your health improved in the ways that you hoped or expected?
      1. Tell me more.
   3. Probe, if needed: has your weight changed in the way you hoped or expected?
      1. Tell me more.

Outcomes and sustainability

1. Tell me about your plans, if any, to stick to this meal plan after the program ends.
   1. Tell me more.
   2. If plans to stick with it, ask: why might you stick with the program?
   3. If plans not to stick with it, as: why not?
   4. Probe, if needed: what might be some challenges, if any, of sticking to this meal plan after the program ends?

Changes to improve the program

*We would like to improve this program to help more people to prevent diabetes.*

1. What, if anything, could have made the low-carbohydrate meal plan easier for you to follow?
   1. Tell me more.
2. What suggestions do you have to improve this program so far?
   1. Tell me more.
   2. Probe, if needed: what changes would you make to help participants to better understand and follow the low-carbohydrate meal plan?
3. Are there particular topics that you would like to cover during the last 6 months of the program?
   1. Tell me more.
4. Would you change anything about the length or frequency of the sessions?
5. This group is designed to run for approximately one year. Tell me what you think about the length of the study?
   1. Did you have concerns about joining a group with that extensive of a commitment?
   2. Would you have been interested if the group was offered for a shorter time period? (length or duration)
      1. Probe, if needed: shorter or longer duration or length?
      2. Probe, if needed: would you change the frequency of meetings?
6. Thinking about once the group stops meeting regularly, what sort of ongoing support, if any, would you like? (informal peer check-ins, participating in future groups as a peer mentor, etc)

Support

*We would like to understand how this program supports participants so that we can develop new ways, if needed, to help more people achieve their health and weight loss goals.*

1. Tell me about the support you received from your lifestyle coach.
   1. Tell me more.
   2. Probe, if needed: was she available, responsive, able to answer questions?
2. Tell me about the support you received from your classmates.
   1. Tell me more.
3. Was there anyone else such as a friend or family member that provided you with support during this program?
   1. Tell me more.
4. Did you speak with your healthcare provider about your participation in this program?
   1. If yes: Tell me about what he or she said or advised.
      1. Probe, if needed: Did you feel supported by your healthcare provider?
5. Are there ways that this program could better support you in achieving your health goals?
   1. Tell me more.

Conclusion

1. Are there any other thoughts or experiences that you would like to share?

I want to thank you again for taking the time to discuss your thoughts and experiences. We will send you a $25 gift card in the mail. This concludes today’s interview. Thank you and goodbye. **Turn off recorder**
